# Outpatient Intravenous Ketamine Protocol for Psychiatric Care at the General Hospital of Corfu

**DOI:** 10.1192/j.eurpsy.2025.864

**Published:** 2025-08-26

**Authors:** M. Demetriou, P. Argitis, M. Peyioti, V. Anagnostopoulou, Z. Chaviaras

**Affiliations:** 1 Psychiatry Department, General Hospital of Corfu, Corfu; 2 University General Hospital of Alexandroupolis, Alexandroupolis, Greece

## Abstract

**Introduction:**

Intravenous (IV) ketamine has garnered increasing attention as a rapid-acting treatment for severe psychiatric conditions such as major depressive disorder (MDD) and suicidal ideation, particularly in treatment-resistant cases. Unlike traditional antidepressants, which often take weeks to exhibit therapeutic effects, IV ketamine can provide relief within hours. While the nasal spray form of ketamine has been approved for clinical use, it remains costly and may not be accessible to all patients. Developing a standardized, cost-effective protocol for the outpatient administration of IV ketamine could enhance treatment accessibility while maintaining safety and efficacy.

**Objectives:**

To develop and evaluate a protocol for the outpatient administration of IV ketamine that ensures patient safety, maximizes clinical effectiveness, and reduces costs compared to alternative ketamine formulations, while maintaining practical outpatient management without requiring inpatient monitoring.

**Methods:**

This study enrolled 46 patients diagnosed with treatment-resistant MDD. Each patient received an initial IV ketamine infusion at a dose of 0.2 mg/kg, followed by 0.5 mg/kg in subsequent sessions, administered over 45 minutes in a 100 mL saline solution. Treatments were performed in an outpatient setting with close monitoring during the infusion and for one hour post-infusion. Symptom improvement was evaluated using standardized psychiatric assessment tools, such as the Montgomery-Åsberg Depression Rating Scale (MADRS) and the Hamilton Depression Rating Scale (HAM-D). The protocol also outlined strategies for managing side effects, including transient dissociation, nausea, and hypertension.

**Results:**

The outpatient ketamine protocol demonstrated rapid and significant symptom relief in the majority of patients, with most reporting improvement after the first or second session. Adverse effects were generally mild, with the most common being transient dissociation and elevated blood pressure, both of which resolved without requiring additional intervention. Importantly, the need for expensive inpatient care or nasal spray formulations was minimized, making the treatment more accessible. The cost savings compared to other ketamine delivery methods were notable, making this protocol a viable option for outpatient psychiatric care.

**Conclusions:**

This study establishes that IV ketamine can be safely and effectively administered in an outpatient setting, offering rapid symptom relief for treatment-resistant MDD while minimizing side effects and reducing overall treatment costs. The protocol presents a practical, cost-effective alternative to more expensive ketamine formulations, providing a feasible solution for broader clinical use in psychiatric outpatient settings. Further research is recommended to validate these findings across larger patient populations and explore long-term outcomes.

**Disclosure of Interest:**

None Declared

